# Intradiscal Condoliase Injection for Lateral Lumbar Disc Herniation in a Patient Receiving Long-Term Hemodialysis: A Case Report

**DOI:** 10.7759/cureus.115422

**Published:** 2026-08-30

**Authors:** Kyohei Nagayama, Yasushi Fujiwara, Kenji Endo, Takato Aihara, Kengo Yamamoto

**Affiliations:** 1 Department of Orthopedic Surgery, Tokyo Medical University, Tokyo, JPN; 2 Department of Orthopedic Surgery, Orthopedic and Microscopic Spine and Spinal Cord Surgery Center, Hiroshima City North Medical Center Asa Citizens Hospital, Hiroshima, JPN

**Keywords:** chemonucleolysis, condoliase, dialysis, lumbar disc herniation, minimally invasive treatment

## Abstract

Spine surgery in dialysis-dependent patients is challenging because of the high risk of perioperative complications. We report the case of a chronic dialysis patient with lateral lumbar disc herniation (LDH) successfully treated with intradiscal condoliase injection therapy. A 51-year-old Japanese male who had been undergoing hemodialysis for 16 years presented with severe left lower extremity pain caused by a left L5-S1 disc herniation with both foraminal and extraforaminal components. Conservative treatments were ineffective, and intradiscal condoliase injection was performed without complications. The patient’s pain significantly improved within three months after the injection. An MRI performed 15 months after treatment demonstrated marked shrinkage of the herniation, and no recurrence of leg pain was observed during the 15-month follow-up period. Condoliase therapy was developed in Japan and has shown favorable outcomes for LDH; however, there have been no previous reports in dialysis-dependent patients. Intradiscal condoliase injection may be a safe and effective minimally invasive treatment option for high-risk dialysis-dependent patients with LDH.

## Introduction

Lumbar disc herniation (LDH) is one of the most common spinal disorders that causes severe lower back pain and leg pain. The lateral-type LDH, which occurs in the foraminal region, accounts for 7% to 12% of all LDHs [1]. The lateral-type LDH compresses the dorsal root ganglion of the exiting nerve root, and it causes more severe leg pain than the central-type LDH. When non-surgical treatments, including medication, nerve root blocks, etc., are not effective, surgical treatment is necessary.

Although surgical treatments for LDH patients can be normally done safely in healthy patients recently, there is difficulty when surgical treatments are necessary for LDH patients undergoing kidney dialysis. Chikuda et al. [2] reported an analysis of a national administrative database in Japan wherein dialysis-dependent patients had a tenfold higher risk of in-hospital deaths than non-dialysis-dependent patients because of major complications such as cardiac events, sepsis, and respiratory complications. Due to the high risk of perioperative complications, minimally invasive treatment is desirable for dialysis-dependent patients with LDH. Chemonucleolysis is a minimally invasive treatment involving the intradiscal injection of an agent that reduces the size of disc herniation and can be performed under local anesthesia without a skin incision [3].

Condoliase was developed in Japan as a novel chemonucleolysis agent and was approved for clinical use in Japan in 2018 [3,4]. Since then, favorable outcomes have been reported for patients with LDH [5-8], including lateral LDH [9]. However, to our knowledge, there have been no previous reports of condoliase treatment in dialysis-dependent patients, although dialysis is not listed as a contraindication in the prescribing information for condoliase. In this report, we describe a dialysis-dependent patient with lateral LDH who was successfully treated with intradiscal condoliase injection therapy.

## Case presentation

A 51-year-old male had asymptomatic lumbar spondylolysis without spondylolisthesis. He had renal failure and had been undergoing hemodialysis for 16 years. He developed low back pain and left lower extremity pain without an obvious trigger and subsequently presented to our clinic in July 2019. Physical examination revealed left L5 radiculopathy. Neurological examination revealed sensory disturbance in the left L5 dermatome, without motor weakness. Plain radiographs showed a pars defect without obvious endplate damage (Figure 1, panel A). Standing flexion-extension radiographs demonstrated no significant segmental instability. 

**Figure 1:**
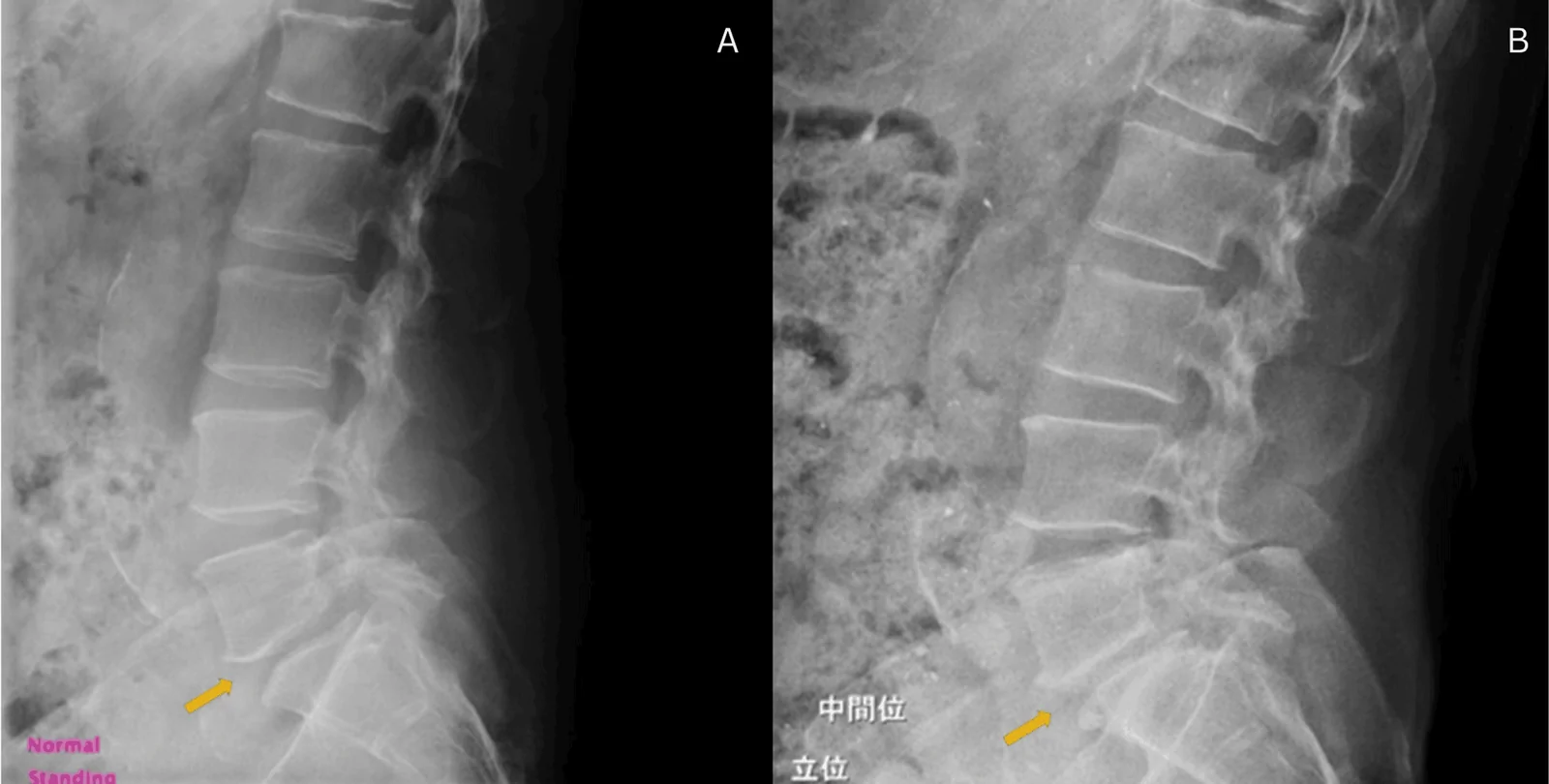
Standing lateral lumbar spine radiographs A: Before the condoliase injection; B: 15 months after the injection; Yellow arrows point to the Pars defects. No obvious vertebral endplate damage is observed on the radiograph.

Axial view of the MRI revealed a left L5-S1 disc herniation with both foraminal and extraforaminal components (Figure 2, panel A). On the sagittal view of the MRI, ventral protrusion of the intervertebral disc and slight endplate changes of the S1 vertebral body were observed (Figure 3, panel A).

**Figure 2:**
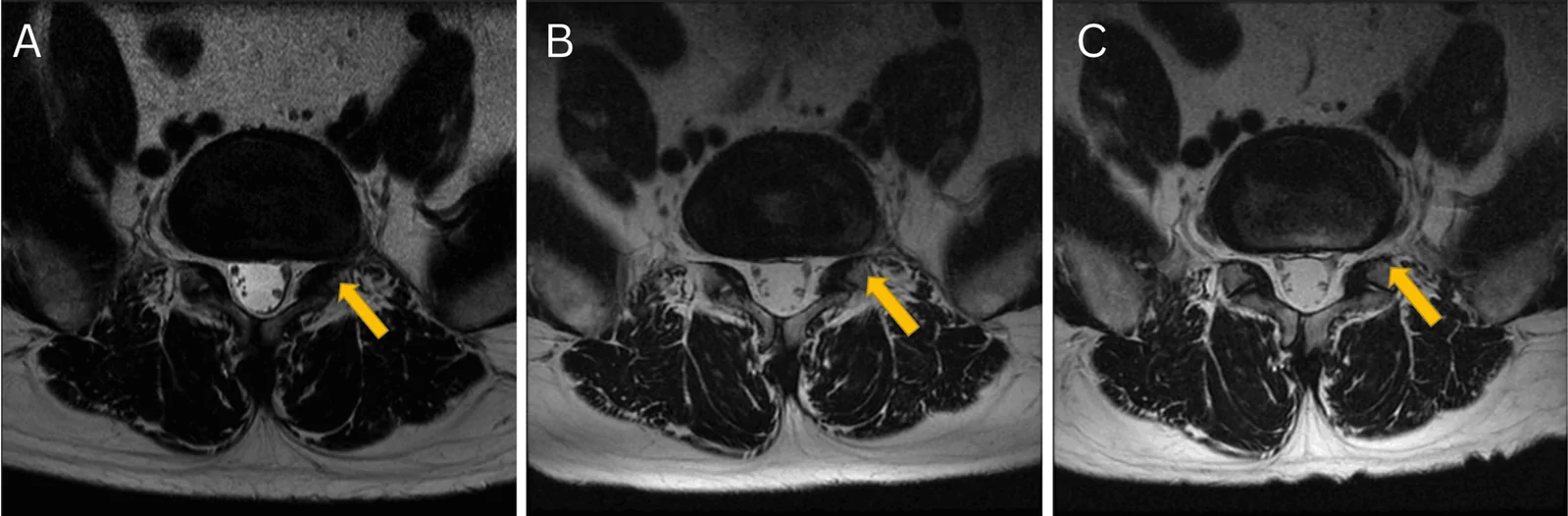
Axial view of MRI A: Before condoliase injection, a left L5-S1 disc herniation with both foraminal and extraforaminal components is observed; B: Three months after condoliase injection; C: 15 months after condoliase injection, the LDH shows a reduction; Yellow arrows point to the LDH. LDH: Lumbar disc herniation

**Figure 3:**
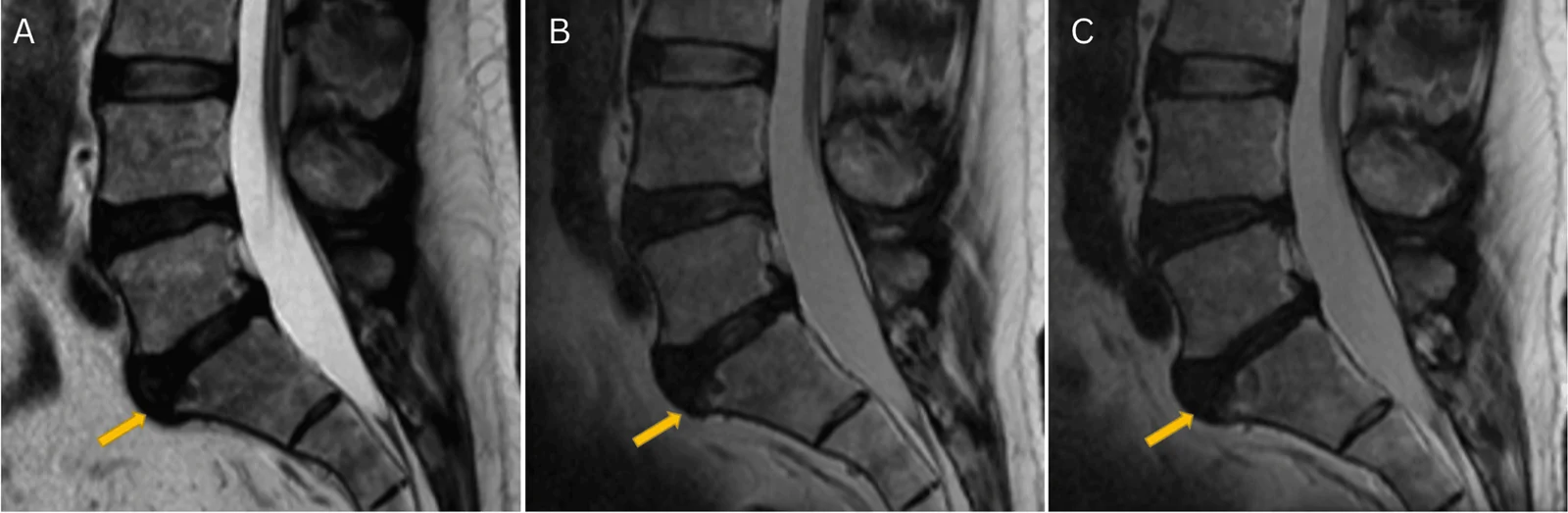
Sagittal view of MRI A: Before condoliase injection, a ventral protrusion of the intervertebral disc and slight endplate changes in the S1 vertebral body are observed; B: Three months after condoliase injection; C: 15 months after condoliase injection, there is no progression of the disc protrusion and endplate change or change in disc height

The patient's symptoms persisted despite six months of conservative treatment, including oral etodolac, limaprost alfadex, pregabalin, and a single selective L5 nerve root block. Therefore, a single intradiscal injection of 1.25 U condoliase (Hernicore® [3]) was performed in a fluoroscopy suite under local anesthesia and fluoroscopic guidance (Figure 4). The procedure was performed on a non-dialysis day. No hospital admission was required, and no complications occurred. After the injection, the patient resumed the same oral medications used before treatment (etodolac, limaprost alfadex, and pregabalin). These medications were gradually reduced as the pain improved and were eventually discontinued.

**Figure 4:**
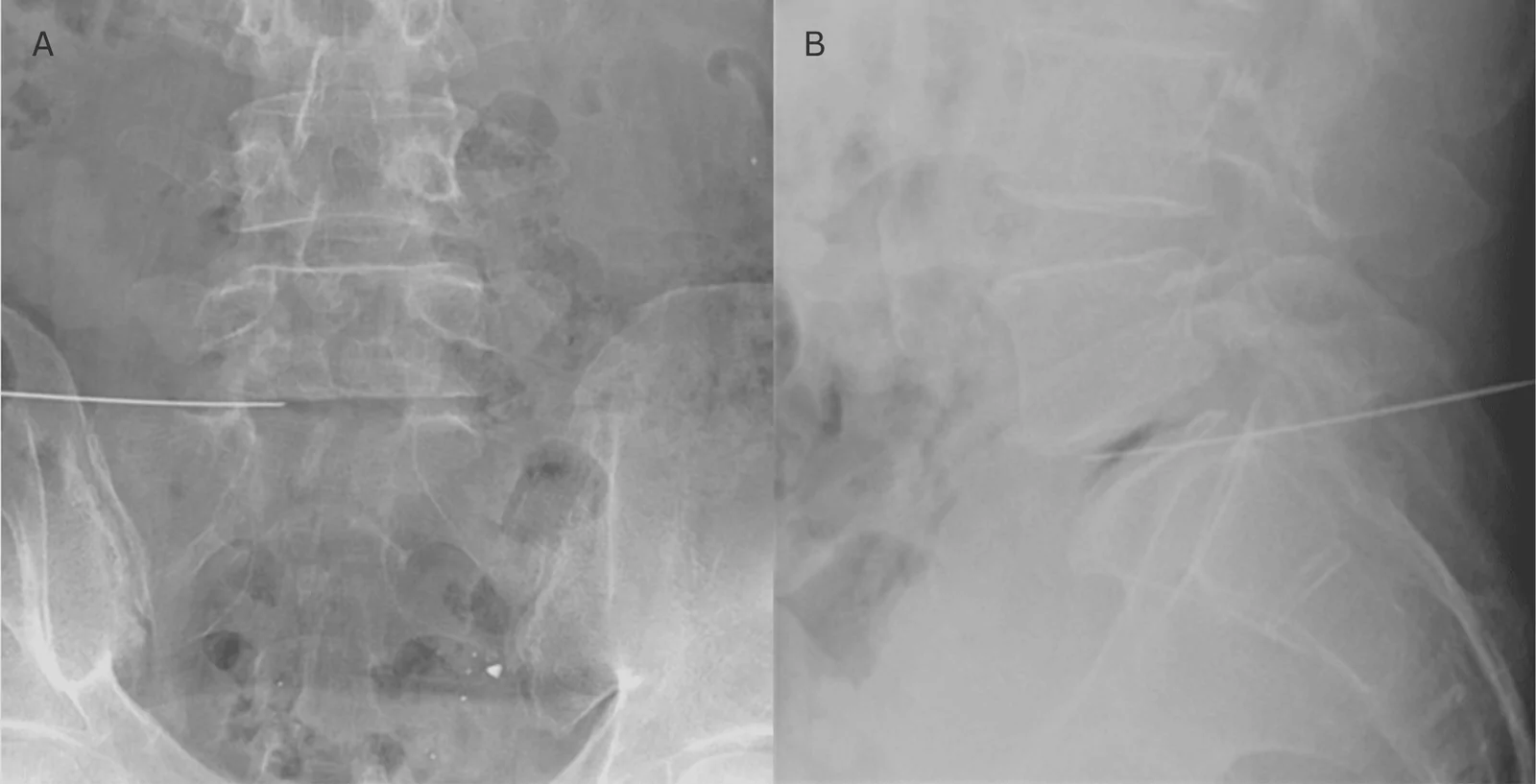
Fluoroscopic images of intradiscal condoliase injection therapy at L5-S1 A: Frontal view; B: Lateral view

The patient's symptoms gradually improved after the injection. Three months post-injection, the VAS scores for low back pain, leg pain, and leg numbness improved from 2.2 to 0.5, 1.8 to 0.6, and 2.0 to 0.5, respectively. The Japanese Orthopaedic Association (JOA) score [10] improved from 19 points before treatment to 26 points at three months and 27 points at six months. The JOA Back Pain Evaluation Questionnaire (JOABPEQ) [11] also demonstrated improvement at three months, particularly in pain-related disability (71.4 to 100), walking ability (21.4 to 78.5), and social functioning (45.9 to 94.5). At six months, the VAS scores for low back pain, leg pain, and leg numbness were 0.6, 0.4, and 0.6, respectively. At approximately one year, the JOA score remained 27 points, and the corresponding VAS scores were 0.8, 0.8, and 0.9, respectively.

The MRI three months after the injection demonstrated shrinkage of the lateral disc herniation (Figure 2, panel B), and the MRI at 15 months showed that the herniation had almost completely disappeared (Figure 2, panel C). Sagittal MRI showed no progression of the disc protrusion or endplate changes (Figure 3, panels B and C). No significant changes in disc height were observed before the injection, at three months, or at 15 months, with measurements of 6.5 mm, 7.0 mm, and 6.7 mm, respectively. At the 15-month follow-up, no recurrence of leg pain was observed, and the patient no longer required oral medications for pain.

## Discussion

This case suggests that chemonucleolysis with intradiscal condoliase injection may be an effective and safe treatment option for lateral LDH in a dialysis-dependent patient. The first chemonucleolysis agent was chymopapain, which has proteolytic activity and reduces the size of disc herniation. Smith et al. first reported its use for chemonucleolysis in 1963 [12], and it was widely used for about 20 years. However, because of its broad proteolytic activity, chymopapain was associated with various complications, including nerve root injury and intervertebral instability [13,14]. Its use was discontinued because of these complications and the risk of anaphylactic reactions.

Condoliase (chondroitin sulfate ABC endolyase) was developed in Japan as a novel chemonucleolysis agent. Condoliase is a pure mucopolysaccharidase derived from the gram-negative rod Proteus vulgaris [4]. It selectively degrades glycosaminoglycans, including chondroitin sulfate, within the nucleus pulposus, with little effect on collagen or other proteins. Therefore, condoliase is considered to be safer than chymopapain. Condoliase was approved for clinical use in Japan in 2018 [4,5], and favorable clinical outcomes have since been reported for LDH [6-8], including lateral LDH [9].

Compared with traditional discectomy, condoliase treatment has several potential advantages. It can be performed under local anesthesia without a skin incision, thereby avoiding the risks associated with general anesthesia and surgery. Moreover, because the procedure can be performed on an outpatient basis or with a short hospital stay, it can be scheduled without substantially disrupting the patient's dialysis schedule.

In the present case, the patient's long-term hemodialysis influenced our treatment strategy. Dialysis-dependent patients have a higher risk of perioperative complications than non-dialysis patients. Therefore, after conservative treatment had failed, we selected intradiscal condoliase injection as a minimally invasive treatment option before considering surgical intervention. In a patient without dialysis, surgical treatment might have been considered earlier depending on the severity of symptoms and imaging findings.

In chronic dialysis patients, destructive spondyloarthropathy characterized by destruction of the vertebral endplates may develop during long-term follow-up, potentially resulting in spinal instability and deformity. Kamimura et al. [15] reported that kyphosis developed rapidly after lumbar decompression surgery in a chronic dialysis patient. Therefore, caution is warranted regarding the potential development of instability or deformity after treatment, and long-term follow-up is important. In this case, the patient was followed up for 15 months. At the final follow-up, symptoms had almost completely resolved, with no evidence of progressive vertebral endplate damage on radiographs or MRI. These findings suggest that intradiscal condoliase injection may reduce disc herniation without causing substantial vertebral endplate damage, although longer follow-up is required.

Although this is only a single case report, further studies involving larger numbers of dialysis-dependent patients and longer follow-up periods are needed to evaluate the long-term safety, efficacy, and appropriate indications for intradiscal condoliase injection. Nevertheless, chemonucleolysis with intradiscal condoliase injection may be a minimally invasive treatment option for dialysis-dependent patients with LDH. Because dialysis-dependent patients are at high risk for perioperative complications, condoliase therapy may be considered as a minimally invasive treatment option before surgical intervention in select patients who have failed conservative treatment and do not exhibit significant spinal instability.

Limitations

This case report has several limitations. First, it describes only a single patient, and the findings may not be generalizable to all dialysis-dependent patients with LDH. Second, the follow-up period was limited to 15 months, and longer-term outcomes remain unknown. Finally, further studies involving larger numbers of dialysis-dependent patients are needed to confirm the safety and efficacy of intradiscal condoliase injection.

## Conclusions

In this dialysis-dependent patient with lateral LDH, intradiscal condoliase injection was associated with sustained clinical improvement. The patient's symptoms improved after treatment, and no recurrence of leg pain was observed during the 15-month follow-up period. The MRI demonstrated regression of the disc herniation without significant radiographic deterioration during follow-up. This case demonstrates favorable clinical and radiographic outcomes following intradiscal condoliase injection in a dialysis-dependent patient with lateral LDH.
